# Wild Caught Nematode Identification and Early Embryo Development: An accessible undergraduate research experience

**DOI:** 10.17912/micropub.biology.000447

**Published:** 2021-08-16

**Authors:** Haley Attix, Alex George, Henali Panchal, Angel Cortez, Martin Cho, Kathy Zarilla, Eric Hastie

**Affiliations:** 1 Durham Technical Community College; 2 University of North Carolina at Chapel Hill

## Abstract

Ample evidence suggests that participation in undergraduate research in community college is critical for stimulating interest and retention in STEM careers. Guided skill development and practice in a collaborative lab setting allows students to be competitive when applying to future research opportunities. The goals of this undergraduate research experience (URE) was for student-driven discovery with unknown outcomes including: introduction to primary literature, developmental biology, developing hypotheses, learning worm maintenance, microscopy, PCR, and sequencing analysis. The use of *C. elegans* and wild caught nematodes facilitated an exciting and affordable project that can be built on in future UREs.

**Figure 1. Comparison of N2 and wild caught nematode early embryo divisions, 18S subunit gDNA amplification and sequencing analysis, with additional morphology comparison for identification:**
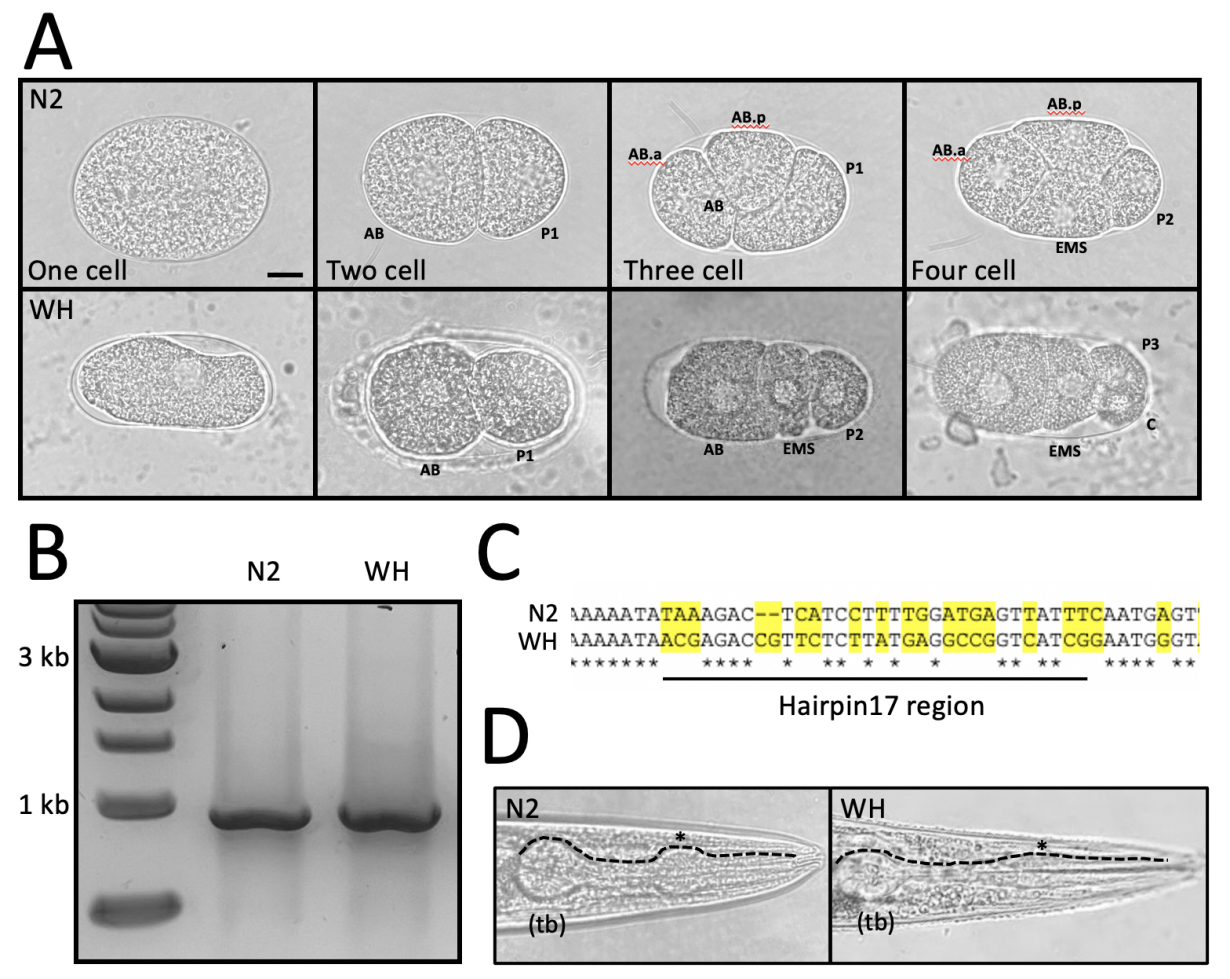
**A. Embryonic cell divisions** of N2 Bristol (common lab strain) and a wild caught Woods Hole (WH) strain. Early embryonic cells are labelled to highlight differences in cell division programs from one through 4 cell stages (Goldstein, 2001). Scale bar is ~10um. **B. Electrophoresis gel of PCR** product run with template gDNA extracted from N2 and WH strains to verify 18S ribosomal subunit band for sequencing (~884 bps). **C. Sequence alignment** of a conserved hairpin 17 region of the 18S sequence of N2 and the WH strain. A BLAST search identified the WH strain as *Acrobeloides* sp. **D. Morphological comparison** of N2 and WH strains shows an expected pronounced terminal bulb (tb) of the adult pharynx in both species. The anterior bulb (*) is noticeable in N2, but less pronounced in the WH strain as seen in previous characterization of *Acrobeloides*.

## Description

Research experiences in community college lead to increased retention in science, technology, engineering, and mathematics (STEM) (Nerio *et al.*, 2019). This two week undergraduate research experience (URE) was designed to enhance laboratory skills in students with limited prior exposure, introduce developmental biology and genetics in a model organism system (*C. elegans*), and encourage participation in generation of data for a micropublication. The University of North Carolina at Chapel Hill and Durham Technical Community College partnered to host the URE for two weeks, for two hours, 4 days a week to limit lab time for students who work full time jobs. Here, we report our findings comparing early developmental cell division of wild type N2 embryos and a wild caught strain that was obtained from soil outside of Loeb Hall in Woods Hole, MA in 2017. The strain, originally called WH strain, was grown on OP50 and survived, suggesting it is a bacteriovore. The WH nematode lays embryos at the one cell stage, making early divisions observable without the dissection or bleaching required for the N2 strain. Students used primers to amplify the 18S ribosomal subunit gene—used in phylogenetic analysis of taxa—from extracted genomic DNA and sent the product for sequencing (Floyd *et al.*, 2005). The hairpin 17 region was selected to display a comparison because of high conservation (Nyaku *et al.*, 2013).BLAST results for the N2 strain matched N2 and results for the wild caught WH strain matched with the nematode strain *Acrobeloides* sp. LKC 27 (a match of 99.7% and E value of 0), available from the Caenorhabditis Genetics Center. LKC 27 was isolated from a western corn rootworm from a Brookings, SD insectary in 2003 (personal communication with Dr. Lynn Carta, USDA-ARS). Students concluded that additional loci need to be examined to determine the relationship of the WH strain to LKC 27.

## Methods

Embryos were extracted from N2 worms using a standard bleaching protocol and genomic DNA was harvested as described in Wormbook. PCR was prepared with MiniOne Taq PCR master mix and run on a miniOne system using an NEB 1kb ladder. Primers for amplification of the 18S subunit gene: 5’ F: AAAGATTAAGCCATGCATG / 5’ R: CATTCTTGGCAAATGCTTTCG, with a predicted product size ~884 kb. PCR conditions: Initial denature of gDNA 94 C, 60 seconds (s) followed by 30 cycles of denature at 94 C for 5s, anneal at 55 C for 10s, and extend for 72 C for 60s , with a final extension at 72 C for 5 minutes and overnight hold at 10 C. PCR products were column purified and sent for sequencing with either primer listed in the reagents section. Embryo images were taken at 100X and adult pharynx images at 40X through the microscope eyepiece with various cell phone cameras. **Worm pick note:** To avoid the high cost of platinum wire, worm picks were made with 32g kanthal A-1 wire (250-foot roll for ~$7 on Amazon) after consultation with an engineer in Dr. Hastie’s family, Dr. L. Wynn Herron. Kanthal picks performed as well as platinum. Stainless steel was also tried, but the heating and cooling created continuous metal flaking.

## Reagents

N2 nematodes were a gift from the Sherwood lab at Duke University and were maintained at room temperature on OP50 plates, a gift from the Gordon lab at UNC-Chapel Hill. WH nematodes were collected from a buried banana sample outside of Loeb Hall in Woods Hole, MA during the 2017 embryology course. Initial time lapse videos were collected by Dr. Leslie Babonis of Cornell University who helped inspire this project.
